# Travel Distance, Urbanicity, and Cardiac Rehabilitation Participation in Medicare Beneficiaries

**DOI:** 10.1016/j.jacadv.2025.102497

**Published:** 2025-12-26

**Authors:** Usman Khan, Jie Yang, Tyler M. Bauer, Maximilian A. Fliegner, Devraj Sukul, Steven J. Keteyian, Robert B. Hawkins, Donald S. Likosky, Michael P. Thompson

**Affiliations:** a Department of Cardiac Surgery, University of Michigan, Ann Arbor, Michigan, USA; b Oakland University William Beaumont School of Medicine, Auburn Hills, Michigan, USA; c Corewell Health, Grand Rapids, Michigan, USA; d Division of Cardiovascular Medicine, Henry Ford Hospital, Detroit, Michigan, USA; e Center for Healthcare Outcomes and Policy, University of Michigan, Ann Arbor, Michigan, USA

**Keywords:** access to care, cardiac rehabilitation, geography, Medicare

## Abstract

**Background:**

Cardiac rehabilitation (CR) is a key intervention for patients recovering from major cardiovascular procedures, but access may be limited.

**Objectives:**

The purpose of this study was to evaluate the relationship between travel distance to the nearest CR facility, degree of urbanicity, and CR participation.

**Methods:**

A retrospective cohort study was conducted on a sample of 100% Medicare fee-for-service claims for beneficiaries with a recent cardiovascular procedure between July 2016 and December 2018. Travel distance between the beneficiary and nearest CR facility was estimated using estimated travel distance between zip codes using Google Maps and categorized as being within the same zip code, 1-15 miles, 16-30 miles, and 30+ miles. Urbanicity was classified as urban, suburban, small town, and rural using U.S. Census data. Multivariable logistic regression compared CR enrollment (attending at least one session) and completion rates (attending 36 sessions or more) across distance and urbanicity categories.

**Results:**

Of the 501,049 beneficiaries in the sample, 76% of urban beneficiaries lived within 15 miles of the nearest CR facility (80,182/84,345), and 63% of rural beneficiaries 15 miles away or more (70,892/112,697). CR enrollment was highest among beneficiaries living within the same zip code as a CR facility (45.6%, 53,935/118,642) and lowest among those living 30 or more miles from the nearest CR facility (19.5%, 7,462/38,309), with an adjusted difference of −22.6 (95% CI: −23.1 to −22.0; *P* < 0.001). In rural areas, the effect of travel distance on CR enrollment and completion was significantly greater when compared with individuals in urban areas.

**Conclusions:**

Increased travel distance was associated with lower overall CR participation, with the effect varying by local degree of urbanicity.

Cardiac rehabilitation (CR) is guideline recommended for improving patient recovery and reducing the risk of future cardiac events following major cardiovascular procedures such as coronary artery bypass grafting (CABG), percutaneous coronary intervention (PCI), and surgical aortic valve replacement (SAVR) or transcatheter aortic valve replacement (TAVR).^1^, ^2^, ^3^, ^4^ Despite guideline recommendations, enrollment rates in CR programs remain low among eligible patients.^5^ Understanding barriers to CR enrollment and completion may identify intervenable targets to improve overall participation.

The travel distance between a patient’s home or place of residence and the nearest CR facility may influence subsequent enrollment and completion to CR, but the relationship has been understudied. Prior research indicates that closer proximity to CR facilities increases enrollment, although these studies have been limited to singular urban areas or regions in the United States or in other countries.^6^^,^^7^ Additionally, rural areas often face challenges such as fewer health care facilities, longer travel distances, and limited health care resources.^8^^,^^9^ Recent studies have highlighted that rural residents have exhibited higher enrollment and completion rates in CR despite fewer available centers, suggesting complex patterns in CR utilization across different regions.^10^ Conversely, urban areas, despite having more health care facilities, are inequitably distributed and may result in significant access disparities, particularly for racial and ethnic minority groups.^6^ Understanding the relationship between travel distance, urbanicity, and CR utilization may inform targeted quality improvement and policy interventions to improve overall CR enrollment.

This study describes the association between travel distance and urbanicity and their interaction with CR enrollment among Medicare beneficiaries who underwent CABG, SAVR, PCI, and TAVR. Specifically, CR enrollment and completion rates were compared across categories of travel distance and urbanicity, while adjusting for patient, hospital, and regional factors. It was hypothesized that the significant interaction between patient distance to the nearest CR facility and the degree of urbanicity would be associated with subsequent CR enrollment and completion.

## Methods

This study was reviewed and approved as human subject protection by the University of Michigan Institutional Review Board (HUM00175541). The authors (M.P.T., J.Y.) had full access to all the data in the study and took responsibility for its integrity and the data analysis. The underlying data in this study cannot be made available due to existing data use agreements.

### Data source and sample

The study used 100% Medicare fee-for-service claims data files from 2016 to 2019, including Medicare Provider Analysis and Review (MedPAR), outpatient facility, Carrier (professional), and Medicare Beneficiary Summary File (MBSF) data. Secondary data sources included the American Hospital Association Annual Survey (2017) for hospital characteristics and the Distressed Community Index (DCI) for regional characteristics. The DCI is a composite measure of local community distress based on the American Community Survey (2014-2018) developed by the Economic Innovation Group, and includes information on zip code–level rates of high school diploma education, poverty, employment, housing vacancy, median household income, change in employment, and change in local establishments.^11^

Beneficiaries were eligible for inclusion in the study if they had 12 months of continuous Part A and B coverage and underwent CABG, PCI, or SAVR or TAVR between July 2016 and December 2018, based on International Classification of Diseases-10th Edition procedure codes in MedPAR data.^12^ For each beneficiary, we identified all CR-eligible procedures and defined the index event as the first qualifying procedure. Procedures within 90 days postdischarge were considered part of the same episode and not counted as new index events; procedures occurring ≥90 days postdischarge were eligible to start a new index event. The initial sample included 547,686 beneficiaries. In this case, the index hospitalization procedure was assigned as their procedure category. Beneficiaries were excluded from the sample if they died during the inpatient stay (n = 18,583), were discharged to hospice (n = 2,803), died within 30 days of discharge (n = 10,117), or were unable to be linked to American Hospital Association or DCI data (n = 15,134). The final analytic sample included 501,049 beneficiaries.

### Cardiac rehabilitation use

The primary outcome in the study was CR enrollment (yes vs no), which was defined as the presence of at least one paid claim for CR within 365 days of discharge. Claims for CR were identified in outpatient facility and Carrier files based on established methods using Current Procedural Terminology codes (93797, 93798) and health care common procedure coding system codes (G0424, G0423 with revenue center code 943).^5^^,^^12^ The secondary outcome in the study was CR completion (yes vs no), which was defined as completing all 36 recommended CR sessions among beneficiaries attending at least one CR session within 365 days of discharge.

### Travel distance and urbanicity

Google Maps was used to quantify the travel distance from the beneficiary to the nearest CR facility locations using geographic centroids of zip codes. We note that the estimated travel distance is not a straight-line distance, but rather the estimated travel distance between the 2 zip code centroids by road. Medicare claims data were used to identify the locations for both the beneficiary and the CR facility. Specifically, the beneficiary zip code was obtained from the MBSF file, and the CR facility zip code was obtained from the outpatient facility file. The estimated travel distance between the beneficiary and CR facility zip code was then grouped into 4 categories: within the same zip code, 1-15 miles, 16-30 miles, and 30+ miles.

Urbanicity was derived from the DCI data source, which uses the U.S. Census urban and rural classification and urban area criteria to classify a zip code as urban, suburban, small town, and rural. Urban was defined as more than 50% of the zip code’s population lives in an urbanized area and the zip code is located in a city with a population of 100,000 or higher. Suburban was defined as more than 50% of the zip code’s population lives in an urbanized area and the zip code is located in a city with a population <100,000. Small town was defined as more than 50% of the zip code’s population lives in an urban cluster, defined as an area of <50,000 people. Rural was defined as more than 50% of the zip code’s population lives in an area classified as rural by the U.S. Census Bureau.

### Covariates

Several patient and hospital covariates were created for this study. Patient age at the time of hospitalization, self-reported sex (male or female), and race/ethnicity (categorized as White, Black, Hispanic, and others including Asian, American Indian/Alaskan Native, Hawaiian/Pacific Islander, multiracial, and unknown/missing), and dual eligibility for Medicare and Medicaid were obtained from the MBSF. MedPAR data were used to identify if the admission was elective or nonelective, if the patient was transferred from another facility during their index hospitalization, the length of hospital stay, and whether the patient was discharged to an extended care facility or home. The Charlson Comorbidity Index was used to assess overall comorbidity level and categorized as a score of 0, 1 to 2, 3 to 4, or 5+.^13^ The DCI was used to categorize a beneficiary’s community-level distress and categorized based on the quintile of DCI score (range: 0-100) according to best practices with the first quintile representing the least distressed communities and fifth quintile representing the most distressed communities.^11^ The claims-based frailty index is a validated measure of frailty and was categorized based on the quartile of frailty score in the sample.^14^ Indicator variables for procedure type were created to indicate if a patient underwent a CABG, PCI, SAVR, or TAVR procedure and could include more than one procedure during the index hospitalization per beneficiary. Hospital characteristics included hospital bed size (categorized as < 100, 100-299, 300-499, or 500+), teaching status (major teaching, minor teaching, or nonteaching), and geographic location by census region (Midwest, Northeast, South, or West).

### Statistical analysis

Univariate and bivariate analyses were used to describe the sample characteristics of the overall sample and stratified by category of distance to the nearest CR facility. Analysis of variance and chi-square tests were used to test for significant group-level differences in patient factors across categories of distance to CR facility. No pairwise post hoc comparisons were conducted. Bivariate analyses compared the sample distribution and crude rates of CR enrollment and completion across categories of distance to nearest CR facility and urbanicity. Multivariable logistic regression models estimated adjusted CR enrollment and completion rates across both CR facility distance and urbanicity categories, adjusting for patient, hospital, and regional factors. Model fit for enrollment and completion were evaluated using the area under the curve estimate with 95% CIs from a bootstrapped sample of 500 replications. Average marginal effects, calculated as predicted probabilities for each individual at their observed covariate values and averaged across the sample, quantified the adjusted absolute difference in CR enrollment and completion rates for beneficiaries living farther from a CR facility compared with those residing in a zip code with a facility (referent), both overall and stratified by urbanicity category. Effect modification between distance and urbanicity categories was tested using a Wald test of the joint significance of the interaction term between the variables. All models were estimated with standard errors clustered at the discharging hospital level to account for within-hospital correlation of observations.

We conducted a number of sensitivity analyses to evaluate the robustness of our findings. First, the analytic sample was restricted to the first qualifying procedure only, thus excluding 6,575 (1.3%) observations for beneficiaries with multiple procedures. Second, travel distance was modeled as a continuous variable and modeled using a linear regression model with the interaction between continuous distance and urbanicity on CR enrollment and completion was evaluated using Wald test of joint significance. To mitigate the impact of outlier data points for distance, the estimated distance variables were Winsorized at the 99th percentile value of distance (66.8 miles). Third, CR completion was redefined as completing 12, 24, and 36 sessions to understand whether the association between distance and completion varied at different points over the course of CR attendance. Finally, an E-value analysis was performed to estimate the robustness of the findings to unmeasured confounding. Because logistic regression yields ORs rather than risk ratios, we fit parallel Poisson regression models with the same model covariates to obtain adjusted risk ratios for CR enrollment and completion, focusing on distance categories within each level of urbanicity.^15^

## Results

### Sample characteristics

Characteristics of the 501,049 beneficiaries in the overall sample and stratified by CR distance category can be found in Table 1. The beneficiaries in the sample were predominantly White, male, with a median age of 75 years, and most were treated in major teaching hospitals or hospitals with more than 500 beds. When comparing characteristics of the sample across categories of distance to nearest CR facility, beneficiaries living further away were more likely to be younger, male, of a non-White race/ethnicity category, and dually eligible for Medicare and Medicaid. There were also significant differences in degree of comorbidity and frailty by distance category.

**Table 1:** Sample Characteristics for the Overall Cohort and Stratified by Category of Travel Distance to the Nearest Cardiac Rehabilitation Facility

	Total	Distance From CR Facility				*P* Value
		Within Zip	0-15 Miles	15-30 Miles	30+ Miles	
Sample size, n (%)	501,049 (100.0%)	118,642 (23.7%)	253,442 (50.6%)	90,656 (18.1%)	38,309 (7.6%)	
Age at admission, median (IQR)	75 (70-81)	75 (70-81)	75 (70-81)	74 (70-80)	74 (70-80)	<0.001
Female, n (%)	185,225 (37.0%)	44,928 (37.9%)	94,471 (37.3%)	32,193 (35.5%)	13,633 (35.6%)	<0.001
Race/ethnicity, n (%)						
White	447,550 (89.3%)	108,354 (91.3%)	221,304 (87.3%)	83,582 (92.2%)	34,310 (89.6%)	<0.001
Black	25,819 (5.2%)	5,175 (4.4%)	15,617 (6.2%)	3,450 (3.8%)	1,577 (4.1%)	
Other	6,759 (1.3%)	1,214 (1.0%)	4,358 (1.7%)	843 (0.9%)	344 (0.9%)	
Asian	6,244 (1.2%)	985 (0.8%)	4,616 (1.8%)	511 (0.6%)	132 (0.3%)	
Hispanic	5,379 (1.1%)	893 (0.8%)	3,214 (1.3%)	706 (0.8%)	566 (1.5%)	
North American native	2,751 (0.5%)	488 (0.4%)	585 (0.2%)	595 (0.7%)	1,083 (2.8%)	
Unknown	6,547 (1.3%)	1,533 (1.3%)	3,748 (1.5%)	969 (1.1%)	297 (0.8%)	
Dual eligible, n (%)	65,924 (13.2%)	14,793 (12.5%)	33,068 (13.0%)	11,602 (12.8%)	6,461 (16.9%)	<0.001
Urbanicity, n (%)						
Urban	84,345 (16.8%)	16,317 (13.8%)	63,865 (25.2%)	3,497 (3.9%)	666 (1.7%)	<0.001
Suburban	186,217 (37.2%)	38,690 (32.6%)	129,820 (51.2%)	16,120 (17.8%)	1,587 (4.1%)	
Small town	117,790 (23.5%)	51,261 (43.2%)	30,326 (12.0%)	22,590 (24.9%)	13,613 (35.5%)	
Rural	112,697 (22.5%)	12,374 (10.4%)	29,431 (11.6%)	48,449 (53.4%)	22,443 (58.6%)	
Distressed Community Index Category, n (%)						
1st-prosperous	121,383 (24.2%)	19,806 (16.7%)	82,684 (32.6%)	16,672 (18.4%)	2,221 (5.8%)	<0.001
2nd	109,753 (21.9%)	22,127 (18.7%)	65,800 (26.0%)	18,530 (20.4%)	3,296 (8.6%)	
3rd	98,728 (19.7%)	27,166 (22.9%)	46,154 (18.2%)	18,711 (20.6%)	6,697 (17.5%)	
4th	92,853 (18.5%)	28,587 (24.1%)	34,782 (13.7%)	19,627 (21.6%)	9,857 (25.7%)	
5th-distressed	78,332 (15.6%)	20,956 (17.7%)	24,022 (9.5%)	17,116 (18.9%)	16,238 (42.4%)	
Census region, n (%)						
Midwest	120,868 (24.1%)	40,847 (34.4%)	57,868 (22.8%)	18,859 (20.8%)	3,294 (8.6%)	<0.001
Northeast	92,624 (18.5%)	18,186 (15.3%)	64,093 (25.3%)	9,386 (10.4%)	959 (2.5%)	
South	205,487 (41.0%)	44,658 (37.6%)	92,420 (36.5%)	47,909 (52.8%)	20,500 (53.5%)	
West	82,070 (16.4%)	14,951 (12.6%)	39,061 (15.4%)	14,502 (16.0%)	13,556 (35.4%)	
Procedure, n (%)						
CABG	134,611 (26.9%)	31,918 (26.9%)	65,764 (25.9%)	26,033 (28.7%)	10,896 (28.4%)	<0.001
PCI	277,107 (55.3%)	65,809 (55.5%)	139,281 (55.0%)	50,291 (55.5%)	21,726 (56.7%)	
SAVR	46,900 (9.4%)	10,999 (9.3%)	24,101 (9.5%)	8,510 (9.4%)	3,290 (8.6%)	
TAVR	67,071 (13.4%)	15,648 (13.2%)	36,760 (14.5%)	10,457 (11.5%)	4,206 (11.0%)	
Transferred from another facility, n (%)	74,881 (14.9%)	21,460 (18.1%)	29,417 (11.6%)	15,547 (17.1%)	8,457 (22.1%)	<0.001
Elective admission, n (%)	186,679 (37.3%)	44,895 (37.8%)	93,727 (37.0%)	33,619 (37.1%)	14,438 (37.7%)	<0.001
Index length of stay, mean (SD)	5.7 (5.7)	5.6 (5.6)	5.8 (5.8)	5.6 (5.4)	5.6 (5.5)	<0.001
Discharge location, n (%)						
ECF/other	98,869 (19.7%)	24,496 (20.6%)	51,342 (20.3%)	16,280 (18.0%)	6,751 (17.6%)	<0.001
Home	402,180 (80.3%)	94,146 (79.4%)	202,100 (79.7%)	74,376 (82.0%)	31,558 (82.4%)	
Charlson Comorbidity Index category, n (%)						
None	41,593 (8.3%)	9,923 (8.4%)	21,081 (8.3%)	7,567 (8.3%)	3,022 (7.9%)	<0.001
1-2	211,899 (42.3%)	50,286 (42.4%)	105,769 (41.7%)	38,912 (42.9%)	16,932 (44.2%)	
3-4	129,310 (25.8%)	30,563 (25.8%)	65,202 (25.7%)	23,628 (26.1%)	9,917 (25.9%)	
5+	118,247 (23.6%)	27,870 (23.5%)	61,390 (24.2%)	20,549 (22.7%)	8,438 (22.0%)	
Claims-based Frailty Index Quartile, n (%)						
Q1 lowest	128,158 (25.6%)	30,064 (25.3%)	65,593 (25.9%)	23,210 (25.6%)	9,291 (24.3%)	<0.001
Q2	126,550 (25.3%)	29,754 (25.1%)	63,657 (25.1%)	23,277 (25.7%)	9,862 (25.7%)	
Q3	124,730 (24.9%)	29,587 (24.9%)	62,587 (24.7%)	22,767 (25.1%)	9,789 (25.6%)	
Q4 highest	121,611 (24.3%)	29,237 (24.6%)	61,605 (24.3%)	21,402 (23.6%)	9,367 (24.5%)	
Hospital bed size, n (%)						
<100	20,143 (4.0%)	6,268 (5.3%)	6,834 (2.7%)	3,936 (4.3%)	3,105 (8.1%)	<0.001
100-300	143,808 (28.7%)	37,788 (31.9%)	63,253 (25.0%)	29,790 (32.9%)	12,977 (33.9%)	
300-500	146,015 (29.1%)	32,870 (27.7%)	76,558 (30.2%)	25,526 (28.2%)	11,061 (28.9%)	
500+	191,083 (38.1%)	41,716 (35.2%)	106,797 (42.1%)	31,404 (34.6%)	11,166 (29.1%)	
Hospital teaching status, n (%)						
Major	134,845 (26.9%)	29,202 (24.6%)	80,652 (31.8%)	19,059 (21.0%)	5,932 (15.5%)	<0.001
Minor	272,274 (54.3%)	64,475 (54.3%)	131,825 (52.0%)	51,885 (57.2%)	24,089 (62.9%)	
Nonteaching	93,930 (18.7%)	24,965 (21.0%)	40,965 (16.2%)	19,712 (21.7%)	8,288 (21.6%)	

CABG = coronary artery bypass grafting; CR = cardiac rehabilitation; ECF = extended care facility; PCI = percutaneous coronary intervention; Q = quartile; SAVR = surgical aortic valve replacement; TAVR = transcatheter aortic valve replacement.

**Figure 1:**
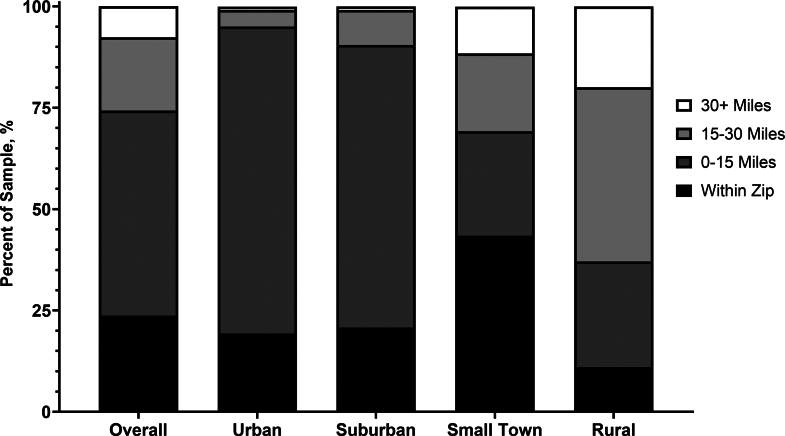
Sample Distribution of Travel Distance and Urbanicity

Among all 501,049 Medicare beneficiaries in the sample, 118,642 (23.7%) had a CR facility within their zip code, 253,442 (50.6%) had one within 15 miles, 90,656 (18.1%) had one between 15 and 30 miles away, and 38,309 (7.7%) had one 30 or more miles away (Figure 1). When stratified by urbanicity categories, 95% of beneficiaries in urban areas have a CR facility within at least 0 to 15 miles or within the same zip code (80,182/84,345), whereas 63% of beneficiaries in rural areas are 15 to 30 miles or 30+ miles away (70,892/112,697).

### Travel distance, urbanicity, and CR enrollment and completion

**Figure 2:**
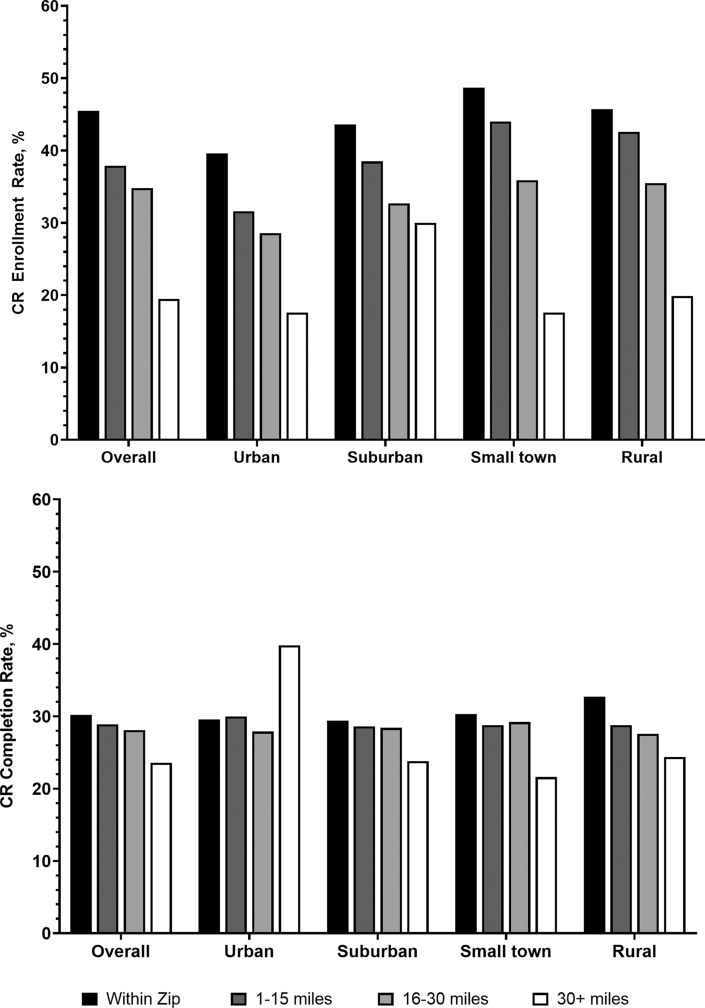
Cardiac Rehabilitation Measure Rates by Travel Distance and Urbanicity CR = cardiac rehabilitation.

Crude rates of CR enrollment by categories of distance to the nearest CR facility, stratified by urbanicity, are shown in Figure 2. Across all groups, there is a clear relationship between proximity to a CR facility and the likelihood of enrollment. Enrollment is highest (45.5%, 53,935/118,642) for beneficiaries living within the same zip code as a CR facility and steadily decreases to 19.5% for those living 30+ miles away from the nearest facility (7,462/38,309). When stratified by urbanicity, enrollment rates were highest for patients in small towns with a CR facility in their zip code (48.7%, 24,943/51,261), while the lowest rates were observed for beneficiaries in urban areas located 30+ miles away from a CR facility (17.6%, 117/666).

**Figure 3:**
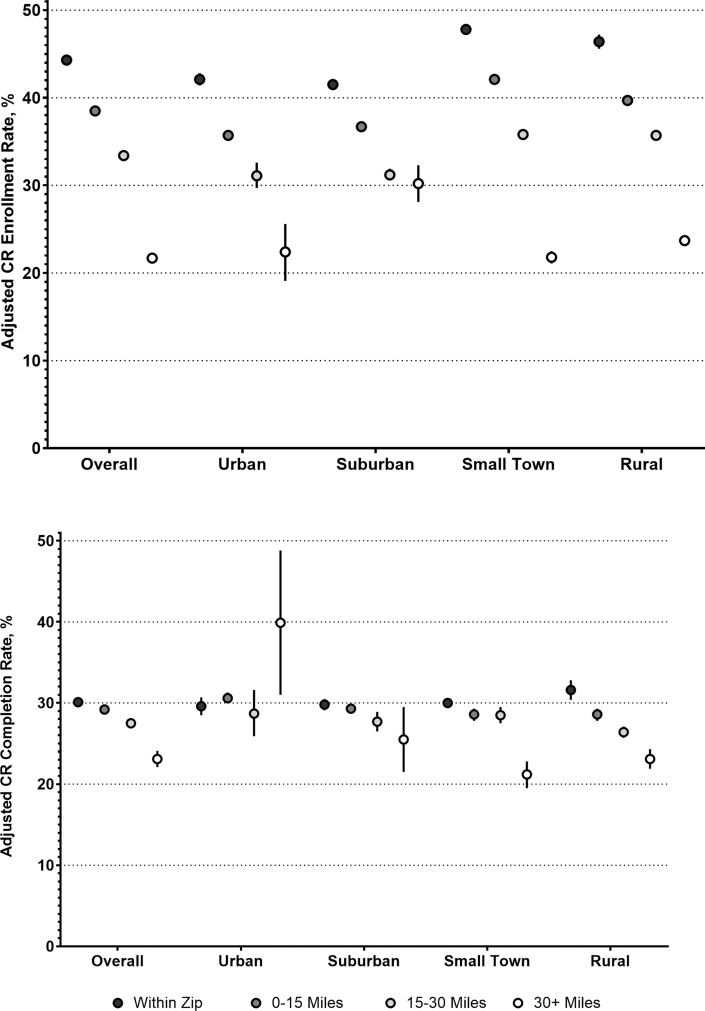
Adjusted Cardiac Rehabilitation Measure Rates and 95% CIs by Travel Distance and Urbanicity Abbreviation as in Figure 2.

Adjusted rates of CR enrollment by travel distance category and urbanicity can be found in Figure 3. Small-town beneficiaries had the highest adjusted rates of CR enrollment but also the largest distance-related gap, with an absolute difference in enrollment of −26.0% (95% CI: −23.7% to −21.7%; *P* < 0.001) when comparing beneficiaries living 30+ miles away to those living in the same zip code as a CR facility (Table 2). Conversely, beneficiaries in suburban areas had the smallest difference in enrollment between beneficiaries living 30+ miles away to those living in the same zip code as a CR facility (adjusted difference = −11.3%; 95% CI: −13.5% to −9.2%; *P* < 0.001). The Wald test of the joint significance of the interaction terms between distance and urbanicity was statistically significant (chi-square = 7,558.02, df = 15; *P* < 0.001) supporting effect modification.

**Table 2:** Adjusted Absolute Differences in Rates of Cardiac Rehabilitation Enrollment and Completion

CR Measure	Urbanicity	Distance to CR Facility						
		Within Zip	1-15 Miles		16-30 Miles		30+ Miles	
		Difference (95% CI)	Difference (95% CI)	*P* Value	Difference (95% CI)	*P* Value	Difference (95% CI)	*P* Value
Enrollment	Overall	Ref	−5.8 (−6.1 to −5.4)	<0.001	−10.8 (−11.3 to −10.4)	<0.001	−22.6 (−23.1 to −22.0)	<0.001
	Urban	Ref	−6.4 (−7.2 to −5.6)	<0.001	−10.9 (−12.5 to −9.2)	<0.001	−19.7 (−23.0 to −16.4)	<0.001
	Suburban	Ref	−4.8 (−5.3 to −4.3)	<0.001	−10.3 (−11.1 to −9.5)	<0.001	−11.3 (−13.5 to −9.2)	<0.001
	Small town	Ref	−5.7 (−6.4 to −5.1)	<0.001	−12.0 (−12.7 to −11.3)	<0.001	−26.0 (−26.9 to −25.2)	<0.001
	Rural	Ref	−6.7 (−7.6 to −5.7)	<0.001	−10.7 (−11.6 to −5.7)	<0.001	−22.7 (−23.7 to −21.7)	<0.001
Completion	Overall	Ref	−0.8 (−1.3 to −0.3)	0.002	−2.6 (−3.3 to −1.9)	<0.001	−7.0 (−8.0 to −5.9)	<0.001
	Urban	Ref	1.0 (−0.3 to 2.3)	0.125	−0.9 (−4.0 to −2.3)	0.556	10.3 (1.3-19.3)	0.025
	Suburban	Ref	−0.5 (−1.3 to 0.2)	0.178	−2.1 (−3.5 to −0.7)	0.003	−4.3 (−8.3 to −0.2)	0.039
	Small town	Ref	−1.5 (−2.4 to −0.5)	0.003	−1.5 (−2.6 to −0.4)	0.010	−8.8 (−10.6 to −7.1)	<0.001
	Rural	Ref	−3.1 (−4.5 to −1.6)	<0.001	−5.2 (−6.6 to −3.8)	<0.001	−8.5 (−10.2 to −6.8)	<0.001

CCI = Charlson Comorbidity Index, CFI = Claims-based Frailty Index; DCI = Distressed Community Index; Ref = referent group; other abbreviation as in Table 1.

Models adjusted for age, sex, race/ethnicity, dual eligibility, procedure type, elective admission, transfer status, index hospital length of stay, discharge to home, CCI category, DCI quintile, CFI quartile, and hospital bed size, teaching status, and census region.

Completion rates, or the percentage of patients completing all 36 recommended CR sessions, are presented in Figure 2. Completion was significantly lower for only those at 30+ miles from the nearest CR facility (23.6%, 1,762/7,462) compared to those within the same zip code (30.2%, 8,875/31,571), 1-15 miles away (28.9%, 27,774/95,995), or 16-30 miles away (28.1%, 16,268/53,935). In adjusted analyses, CR completion was stable within 30 miles but declined sharply beyond this distance across all groups except urban areas, where distant patients had the highest completion rates (based on small numbers). Suburban, small-town, and rural patients showed progressively lower completion with increasing distance, with the lowest rates among small-town patients living ≥30 miles away. The Wald test of the joint significance of the interaction terms between distance and urbanicity was statistically significant (chi-square = 284.21, df = 15; *P* < 0.001) supporting effect modification.

Both models for CR enrollment and completion demonstrated acceptable discrimination with an area under the curve in the enrollment model of 0.747 (95% CI: 0.746-0.748) and completion model of 0.701 (95% CI: 0.699-0.703). Testing for collinearity in both models, no variable had a variance inflation factor >10 and most were under 5 suggesting no evidence of problematic collinearity (Supplemental Table 1).

When the analytic sample was restricted to beneficiaries’ first qualifying procedure only, the findings did not change substantively (Supplemental Table 2). When modeled as a continuous variable, there was a linear relationship between CR distance and predicted probability of CR enrollment and completion across urbanicity categories (Supplemental Figures 1 and 2). Adjusted for covariates, a 1-mile increase in estimated travel distance was associated with a −0.53-percentage point decrease in CR enrollment (95% CI: −0.55 to −0.51; *P* < 0.001) and a −0.15-percentage point decrease in CR completion (95% CI: −0.18 to −0.12; *P* < 0.001), and the effects of distance across urbanicity category were qualitatively similar when compared to the model using distance as a categorical variable (Supplemental Table 3). There was a significant interaction between continuous travel distance and CR enrollment (chi-square = 7,948.01, df = 7; *P* < 0.001) and CR completion (chi-square = 316.26, df = 7; *P* < 0.001).

When redefining completion to be attending 12, 24, and 36 sessions, we found that the patterns largely remain the same, with increasing distance associated with lower completion rates of 12, 24, and 36 sessions, and slightly smaller effects of urbanicity within the same distance category (Supplemental Table 4). Similar to the main findings, those in small town and rural locations with the largest distances to nearest CR facility (16-30 miles or 30+ miles) have the lowest completion rates. The E-value analysis showed our findings comparing distance categories of 30+ miles vs within-zip were relatively robust, with E-values of 3 to 4 for CR enrollment and ∼2 for completion (Supplemental Table 5). However, comparisons of shorter distances, where our effect sizes were smaller, were more susceptible to unmeasured confounding for both enrollment and completion with E-values of ∼1.2 to 1.6.

## Discussion

The objective of this study was to describe the association between travel distance and urbanicity and their interaction with CR participation among Medicare beneficiaries undergoing CR-eligible cardiovascular procedures, and the study has 2 significant findings (Central Illustration). First, travel distance is an important barrier to CR participation, with increased distance associated with significantly lower rates of enrollment. Enrollment rates declined by as much as 22 percentage points for patients that were 30 or more miles away from the nearest CR facility, compared to having one in their own zip code. Completion rates were also significantly lower as travel distance increased, although the magnitude of the effect was smaller relative to enrollment rates. Second, the relationship between travel distance and CR participation varied as a function of the urbanicity in which a beneficiary resides. In rural areas, the effect of travel distance on CR enrollment and completion was significantly greater when compared to individuals in urban areas. However, most beneficiaries in urban areas live within 15 miles of a CR facility, while most beneficiaries in rural areas live 16 or more miles away from the nearest CR facility.

**Central Illustration:**
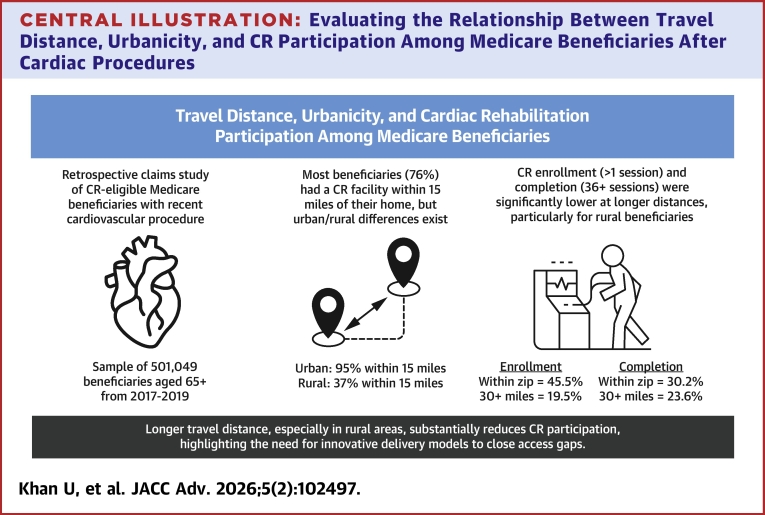
Evaluating the Relationship Between Travel Distance, Urbanicity, and CR Participation Among Medicare Beneficiaries After Cardiac Procedures Abbreviation as in Figure 2.

The main finding from this study is consistent with previous research demonstrating that geographic distance between the patient and CR facility is a significant predictor of CR participation.^7^^,^^9^^,^^16^ As distance to a CR facility increases, both enrollment and completion rates decline, with the decline in enrollment being much stronger than that in completion. Interestingly, the overall effect is most pronounced in urban areas, where the majority of patients (nearly 75%) live within 15 miles of a CR facility. The association between increased distance and reduced enrollment and completion is clear: patients living closer to CR facilities are more likely to enroll, and those living farther away, particularly beyond 30 miles, are less likely to adhere to the program. For the overall population, the impact of distance is significant, but it is mitigated for most, as a large portion of beneficiaries reside within the 15-mile range of a CR facility.

The present study also adds context to the understanding of the effect of geographic distance and CR participation by evaluating the relationship across levels of urbanicity in which the patient resides. Existing literature has demonstrated that urban areas tend to have lower rates of CR enrollment compared to rural areas, but have not investigated how travel distance impacts this relationship.^9^^,^^10^ Our findings suggests that the greatest distances to CR facilities are observed in small-town and rural areas, where distance plays an even more significant role in reducing both enrollment and completion. In these areas, a larger proportion of people live more than 15 miles from a CR facility, making geographic distance a much larger barrier compared to suburban populations, where most residents live closer to CR facilities. This suggests that addressing issues of access to care may be more critical for rural and small-town populations than for suburban patients. The implications of these findings emphasize the need to develop targeted interventions to overcome geographic barriers in rural and small-town populations, such as increasing the availability of CR facilities or exploring alternative methods like virtual CR programs. As the burden of cardiovascular disease and subsequent mortality continues to grow more quickly in rural areas, addressing barriers to CR in these populations may become an even greater priority.^17^^,^^18^

While distance strongly affects CR enrollment, its impact on completion is less consistent, with the most substantial effects observed primarily at extreme distances. Interestingly, urban patients living more than 30 miles away exhibit relatively high completion once enrolled, possibly due to better access to public transportation options, family or community support, and a broader availability of health resources that may ease the burden of travel.^15^ In contrast, rural and small-town patients face greater challenges with completion as distance grows, often due to fewer transportation options, higher travel costs, and the difficulty of making frequent trips to CR facilities located far from their communities.^6^^,^^10^ For these patients, the demands of attending multiple sessions can make program completion nearly impossible, particularly when physical limitations or comorbidities are present. This pattern highlights the need for nontraditional strategies, such as virtual CR programs, especially for rural populations where distance is a significant and consistent barrier to ongoing participation.^19^ For both CR enrollment and completion, there are legislative opportunities to expand access through virtual-based CR options that may disproportionately help rural patients that are often located in areas without CR facilities.^8^^,^^20^

### Study Limitations

These findings should be viewed in light of certain limitations. First, the variable of travel distance is estimated as the road-based travel distance between 2 zip code centroids and may not reflect real distances experienced by patients that live within specific zip codes. While these are reasonable estimates when comparing differences between urban and rural populations they may still misclassify distance traveled for some individuals.^21^ Second, this study does not account for specific factors such as traffic conditions, seasonal variations, or weather impacts that could play a role in access to CR, particularly in urban settings where congestion or adverse weather might influence travel times and attendance.^7^ The 30+ mile distance category also includes fewer patients, which could make it harder to generalize these findings to the broader Medicare population, especially in regions with distinct transportation or health care infrastructure. Third, the observational design restricts our ability to make causal claims, as we cannot determine if the relationships observed are directly caused by distance or urbanicity alone. Furthermore, since this study focuses specifically on patients who underwent CR-eligible procedures, the results may not fully represent patients managed medically for conditions like myocardial infarction, which might face different barriers to CR enrollment. Fourth, our data are limited to observations occurring prior to the COVID-19 pandemic, which has been shown to lead to several CR facility closures.^22^ Future work should evaluate the impact of these closures on travel distance for CR participants. Fifth, while we accounted for clustering using robust standard errors, additional sources of clustering variation may remain unobserved. Hierarchical models could potentially capture this structure more fully, but many clusters in our data had very small sample sizes, limiting the feasibility and stability of multilevel modeling. Finally, while our sample includes procedures that are common qualifying conditions for CR using established methods, we do not include other potentially qualifying events, such as medically managed acute myocardial infarction or heart failure.^12^

In conclusion, geographic distance and urbanicity are significantly associated with both CR enrollment and completion, with the greatest obstacles faced by patients who live in rural and small-town settings and more than 30 miles from a CR facility. These findings highlight the need for focused interventions that can directly address the barriers faced by these populations. Adding more CR facilities in underserved areas implementing virtual CR programs or even exploring mobile CR units could all be effective ways to expand access and improve participation. By implementing these strategies, CR utilization could increase, leading to better recovery outcomes for Medicare beneficiaries and more equitable health care access for those who need it most.

Perspectives **CLINICAL COMPETENCIES:** These findings demonstrate that travel distance and local context significantly influence CR participation, highlighting the need for targeted strategies to improve access and adherence among patients facing geographic barriers.

**TRANSLATIONAL OUTLOOK:** This work advances the scientific continuum by linking population-level evidence on access disparities to the development and implementation of scalable, innovative delivery models to promote equitable cardiovascular care.

## Funding support and author disclosures

Dr Thompson received grant support from the 10.13039/100000133Agency for Healthcare Research and Quality (K01HS027830). Dr Thompson has received salary support from Blue Cross Blue Shield of Michigan for his role in the Michigan Value Collaborative. Dr Sukul has served as a consultant to Angiowave Imaging and RapidAI. Dr Likosky has received research funding from the 10.13039/100000133AHRQ and the 10.13039/100000002National Institutes of Health, is a consultant for the American Society of Extracorporeal Technology, and has received partial salary support from Blue Cross Blue Shield of Michigan to advance quality in Michigan in conjunction with the Michigan Society of Thoracic and Cardiovascular Surgeons Quality Collaborative. Dr Hawkins reports a relationship with Medtronic Inc that includes consulting or advisory. Dr Keteyian is a member of the Scientific Advisory Board for Kento Health. All other authors have reported that they have no relationships relevant to the contents of this paper to disclose.
