# Supplementary material for: Travel Distance, Urbanicity, and Cardiac Rehabilitation Participation in Medicare Beneficiaries
Source: JACC Adv. 2025 Dec 26;5(2):102497. doi: 10.1016/j.jacadv.2025.102497 (PMC12797041; doi:10.1016/j.jacadv.2025.102497)
Supplement: Supplemental_Material [file mmc1.docx]

**Supplemental Table 1. Variance inflation factors for CR enrollment and completion models testing for collinearity.**

| **Variable** | **Category** | **Variance inflation factors** | |
| --- | --- | --- | --- |
|  |  | **Enrollment model** | **Completion Model** |
| Distance Category | Within zip | - | - |
|  | 0-15 | 1.78 | 1.59 |
|  | 15-30 | 1.7 | 1.58 |
|  | 30+ | 1.48 | 1.23 |
| Urbanicity | Urban | - | - |
|  | Suburban | 1.79 | 1.92 |
|  | Small Town | 2.47 | 2.46 |
|  | Rural | 2.05 | 1.89 |
| Age at admissions. | - | 1.2 | 1.19 |
| Male | - | 1.11 | 1.1 |
| Race/ethnicity | White | - | - |
|  | Black | 1.1 | 1.07 |
|  | Other | 1.01 | 1.01 |
|  | Asian | 1.04 | 1.02 |
|  | Hispanic | 1.05 | 1.03 |
|  | NA Native | 1.02 | 1.01 |
|  | Unknown | 1.02 | 1.02 |
| Dual Eligible |  | 1.18 | 1.08 |
| Distressed Community Index Category | 1st-Prosperous | - | - |
|  | 2nd | 1.52 | 1.43 |
|  | 3rd | 1.58 | 1.49 |
|  | 4th | 1.68 | 1.52 |
|  | 5th-Distressed | 1.8 | 1.48 |
| Census Region | Midwest | - | - |
|  | Northeast | 1.59 | 1.4 |
|  | South | 1.7 | 1.44 |
|  | West | 1.61 | 1.4 |
| Procedure | CABG | 6.1 | 5.6 |
|  | PCI | 8.94 | 7.12 |
|  | SAVR | 2.18 | 2.13 |
|  | TAVR | 4.84 | 3.4 |
| Transferred from Another Facility | - | 1.16 | 1.2 |
| Elective Admission | - | 1.75 | 1.85 |
| Index Length of Stay | - | 1.63 | 1.81 |
| Charlson Comorbidity Index Category | Discharge to Home | 1.35 | 1.27 |
|  | 0 | - | - |
|  | 1-2 | 3.68 | 2.92 |
|  | 3-4 | 3.4 | 2.68 |
|  | 5+ | 3.58 | 2.49 |
| Claims-based Frailty Index Quartile | Q1 Lowest | - | - |
|  | Q2 | 1.58 | 1.41 |
|  | Q3 | 1.76 | 1.53 |
|  | Q4 Highest | 2.12 | 1.58 |
| Hospital Bed Size | <100 | - | - |
|  | 100-300 | 6.13 | 5.98 |
|  | 300-500 | 6.5 | 6.16 |
|  | 500+ | 7.99 | 7.77 |
| Hospital Teaching Status | Major | - | - |
|  | Minor | 2.08 | 2.03 |
|  | nonteaching | 2.3 | 2.25 |
|  | Mean VIF | 2.48 | 2.23 |
| Abbreviations: | | | |

**Supplemental Table 2. Adjusted absolute differences in rates of cardiac rehabilitation (CR) enrollment and completion excluding secondary procedures for beneficiaries with more than one procedure.**

| **CR Measure** | **Urbanicity** | **Distance to CR Facility** | | | | | | |
| --- | --- | --- | --- | --- | --- | --- | --- | --- |
|  |  | **Within Zip** | **1-15 miles** | | **16-30 miles** | | **30+ miles** | |
|  |  | **Difference (95% CI)** | **Difference  (95% CI)** | **p-value** | **Difference  (95% CI)** | **p-value** | **Difference  (95% CI)** | **p-value** |
| Enrollment | Overall | Ref | -5.7 (-6.1, -5.4) | <0.001 | -10.9 (-11.4, -10.4) | <0.001 | -18.8 (-19.8, -17.7) | <0.001 |
|  | Urban | Ref | -6.3 (-7.1, -5.5) | <0.001 | -10.8 (-12.5, -9.2) | <0.001 | -19.7 (-23.0, -16.4) | <0.001 |
|  | Suburban | Ref | -4.8 (-5.3, -4.3) | <0.001 | -10.3 (-11.1, -9.5) | <0.001 | -11.5 (-13.7, -9.4) | <0.001 |
|  | Small Town | Ref | -5.7 (-6.4, -5.1) | <0.001 | -12.1 (-12.8, -11.4) | <0.001 | -26.2 (-27.0, -25.4) | <0.001 |
|  | Rural | Ref | -6.7 (-7.7, -5.7) | <0.001 | -10.7 (-11.6, -9.8) | <0.001 | -22.8 (-23.8, -21.8) | <0.001 |
|  |  |  |  |  |  |  |  |  |
| Completion | Overall | Ref | -1.1 (-1.6, -0.5) | <0.001 | -2.5 (-3.3, -1.7) | <0.001 | -4.2 (-6.4, -0.2) | <0.001 |
|  | Urban | Ref | 1.1 (-0.2, 2.3) | 0.107 | -1.1 (-4.2, 2.0) | 0.483 | 9.7 (0.7, 18.7) | 0.034 |
|  | Suburban | Ref | -0.5 (-1.3, 0.3) | 0.196 | -2.1 (-3.5, -0.7) | 0.003 | -4.1 (-8.2, 0.04) | 0.052 |
|  | Small Town | Ref | -1.5 (-2.4, -0.5) | 0.003 | -1.5 (-2.7, -0.4) | 0.009 | -8.7 (-10.5, -7.0) | <0.001 |
|  | Rural | Ref | -3.0 (-4.5, -1.6) | <0.001 | -5.2 (-6.6, -3.8) | <0.001 | -8.6 (-10.3, -6.9) | <0.001 |
| Abbreviations: CR = cardiac rehabilitation, CI = confidence interval, Ref = referent group  Models adjusted for age, sex, race/ethnicity, dual eligibility, procedure type, elective admission, transfer status, index hospital length of stay, discharge to home, CCI category, DCI quintile, CFI quartile, and hospital bed size, teaching status, and census region. | | | | | | | | |

**Supplemental Figure 1. Locally Weighted Scatterplot Smoothing (LOWESS) curves for the crude relationship between continuous estimated travel distance and probability of CR enrollment stratified by urbanicity category.**

**
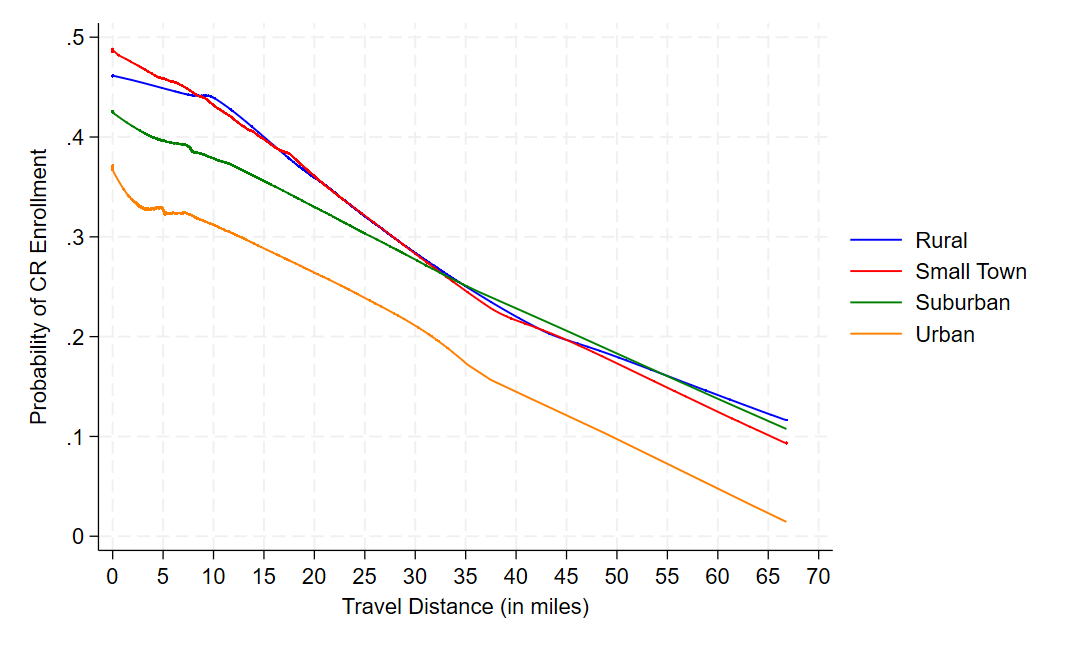
**

**Supplemental Figure 2. Locally Weighted Scatterplot Smoothing (LOWESS) curves for the crude relationship between continuous estimated travel distance and probability of CR completion stratified by urbanicity category.**

**
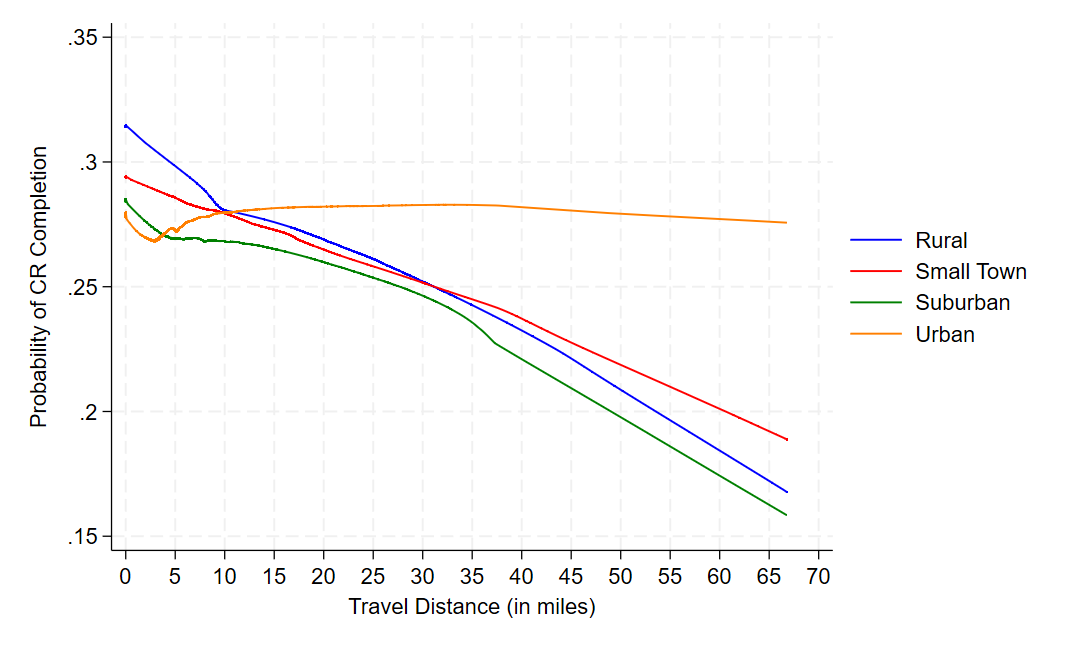
**

**Supplemental Table 3. Adjusted change in CR participation per 1-mile increase in estimated travel distance for the overall sample and by urbanicity category.**

| **CR Measure** | **Urbanicity** | **Difference  (95% CI)** | **p-value** |
| --- | --- | --- | --- |
| CR Enrollment | Overall | -0.53 (-0.55, -0.51) | <0.001 |
|  | Urban | -0.55 (-0.61, -0.49) | <0.001 |
|  | Suburban | -0.49 (-0.52, -0.46) | <0.001 |
|  | Small Town | -0.59 (-0.61, -0.58) | <0.001 |
|  | Rural | -0.52 (-0.54, -0.50) | <0.001 |
|  |  |  |  |
| CR Completion | Overall | -0 15 (-0.18, -0.12) | <0.001 |
|  | Urban | 0.05 (-0.06, 0.16) | 0.381 |
|  | Suburban | -0.16 (-0.22, -0.10) | <0.001 |
|  | Small Town | -0.16 (-0.20, -0.13) | <0.001 |
|  | Rural | -0.23 (-0.27, -0.19) | <0.001 |
| Abbreviations: CR = cardiac rehabilitation, CI = confidence interval | | | |

**Supplemental Table 4. Adjusted rates and differences in CR completion defined as 12, 24, or 36 sessions across categories of travel distance and urbanicity.**

| **Sessions Attended** | **Urbanicity** | **Distance to CR Facility** | | | | | | | | | | |  |
| --- | --- | --- | --- | --- | --- | --- | --- | --- | --- | --- | --- | --- | --- |
|  |  | **Within Zip** | | **1-15 miles** | | | **16-30 miles** | | | **30+ miles** | | |  |
|  |  | **Rate (%)** | **Difference**  **(95% CI)** | **Rate (%)** | **Difference**  **(95% CI)** | **p-value** | **Rate (%)** | **Difference**  **(95% CI)** | **p-value** | **Rate (%)** | **Difference**  **(95% CI)** | **p-value** |  |
| 12 sessions | Overall | 82.9 | Ref | 81.8 | -1.1 (-1.6, -0.7) | <0.001 | 80.3 | -2.6 (-3.3, -1.9) | <0.001 | 76.4 | -6.5 (-8.3, -4.7) | <0.001 |  |
|  | Urban | 82.2 | Ref | 82.4 | 0.1 (-0.9, 1.1) | 0.853 | 82.0 | -0.3 (-2.8, 2.2) | 0.812 | 86.3 | 4.1 (-1.9, 10.0) | 0.178 |  |
|  | Suburban | 82.6 | Ref | 81.7 | -0.9 (-1.6, -0.3) | 0.006 | 79.9 | -2.8 (-4.0, -1.5) | <0.001 | 76.4 | -6.2 (-9.9, -1.5) | 0.001 |  |
|  | Small Town | 83.8 | Ref | 81.8 | -2.0 (-2.8, -1.2) | <0.001 | 80.3 | -3.5 (-4.5, -2.5) | <0.001 | 73.3 | -10.5 (-12.3, -8.7) | <0.001 |  |
|  | Rural | 82.7 | Ref | 81.4 | -1.2 (-2.5, -0.1) | 0.033 | 79.7 | -3.0 (-4.2, -1.9) | <0.001 | 73.1 | -9.6 (-11.2, -8.0) | <0.001 |  |
|  |  |  |  |  |  |  |  |  |  |  |  |  |  |
| 24 Sessions | Overall | 63.5 | Ref | 61.7 | -1.8 (-2.3, -1.2) | <0.001 | 59.4 | -4.1 (-5.0, -3.2) | <0.001 | 55.1 | -8.4 (-10.6, -6.2) | <0.001 |  |
|  | Urban | 62.3 | Ref | 62.8 | 0.5 (-0.8, 1.8) | 0.466 | 61.6 | -0.7 (-4.0, 2.5) | 0.659 | 70.3 | 8.0 (-0.2, 16.1) | 0.055 |  |
|  | Suburban | 64.6 | Ref | 62.5 | -2.2 (-3.0, -1.4) | <0.001 | 58.8 | -5.8 (-7.3, -4.3) | <0.001 | 56.0 | -8.6 (-13.1, 4.1) | <0.001 |  |
|  | Small Town | 63.8 | Ref | 61.2 | -2.5 (-3.6, -1.5) | <0.001 | 60.5 | -3.3 (-4.5, -2.1) | <0.001 | 49.0 | -14.8 (-16.8, -12.7) | <0.001 |  |
|  | Rural | 62.0 | Ref | 60.3 | -1.7 (-3.2, -0.2) | 0.028 | 57.6 | -4.3 (-5.8, -2.9) | <0.001 | 50.3 | -11.7 (-13.6, -9.8) | <0.001 |  |
|  |  |  |  |  |  |  |  |  |  |  |  |  |  |
| 36 Sessions (Completion) | Overall | 30.2 | Ref | 29.1 | -1.1 (-1.6, -0.5) | <0.001 | 27.8 | -2.5 (-3.3, -1.7) | <0.001 | 26.0 | -4.2 (-6.4, -0.2) | <0.001 |  |
|  | Urban | 29.6 | Ref | 30.6 | 1.1 (-0.2, 2.3) | 0.107 | 28.7 | -1.1 (-4.2, 2.0) | 0.483 | 39.9 | 9.7 (0.7, 18.7) | 0.034 |  |
|  | Suburban | 29.8 | Ref | 29.3 | -0.5 (-1.3, 0.3) | 0.196 | 27.7 | -2.1 (-3.5, -0.7) | 0.003 | 25.5 | -4.1 (-8.2, 0.04) | 0.052 |  |
|  | Small Town | 31.0 | Ref | 28.6 | -1.5 (-2.4, -0.5) | 0.003 | 28.5 | -1.5 (-2.7, -0.4) | 0.009 | 21.2 | -8.7 (-10.5, -7.0) | <0.001 |  |
|  | Rural | 31.6 | Ref | 28.6 | -3.0 (-4.5, -1.6) | <0.001 | 26.4 | -5.2 (-6.6, -3.8) | <0.001 | 23.1 | -8.6 (-10.3, -6.9) | <0.001 |  |
| Abbreviations: CR = cardiac rehabilitation, CI = confidence interval  Adjusted for age, sex, race/ethnicity, dual eligibility, procedure type, elective admission, transfer status, index hospital length of stay, discharge to home, CCI category, DCI quintile, CFI quartile, and hospital bed size, teaching status, and census region. | | | | | | | | | | | | | |

**Supplemental Table 5. E-value analysis for unmeasured confounding.**

| **Urbanicity** | **Distance** | **CR Enrollment** | | **CR Completion** | |
| --- | --- | --- | --- | --- | --- |
|  |  | **Estimated RR** | **E-value** | **Estimated RR** | **E-value** |
| Urban | Within zip | Ref | - | Ref | - |
|  | 0-15 miles | 0.843 | 1.66 | 1.034 | 1.22 |
|  | 15-30 miles | 0.737 | 2.05 | 0.969 | 1.21 |
|  | 30+ miles | 0.501 | 3.41 | 1.364 | 2.07 |
| Suburban | Within zip | Ref | - | Ref | - |
|  | 0-15 miles | 0.889 | 1.5 | 0.982 | 1.15 |
|  | 15-30 miles | 0.758 | 1.97 | 0.931 | 1.36 |
|  | 30+ miles | 0.724 | 2.11 | 0.854 | 1.62 |
| Small Town | Within zip | Ref | - | Ref | - |
|  | 0-15 miles | 0.876 | 1.54 | 0.951 | 1.28 |
|  | 15-30 miles | 0.746 | 2.02 | 0.951 | 1.28 |
|  | 30+ miles | 0.429 | 4.09 | 0.707 | 2.18 |
| Rural | Within zip | Ref | - | Ref | - |
|  | 0-15 miles | 0.852 | 1.63 | 0.905 | 1.45 |
|  | 15-30 miles | 0.766 | 1.94 | 0.838 | 1.67 |
|  | 30+ miles | 0.488 | 3.52 | 0.736 | 2.06 |
| Abbreviations: CR = cardiac rehabilitation, RR = relative rate, Ref = referent group | | | | | |
